# Aortic Dissection: An Unexpected Complication of Transcatheter Aortic Valve Implantation

**DOI:** 10.7759/cureus.78607

**Published:** 2025-02-06

**Authors:** André Cabrita, Catarina A Marques, Mariana Vasconcelos, Rui A Rodrigues

**Affiliations:** 1 Cardiology, Unidade Local de Saúde (ULS) São João, Porto, PRT

**Keywords:** acute heart failure, aortic dissection, chronic heart failure, tavi, tavi complications

## Abstract

A 53-year-old female with a medical history of multiple cardiovascular risk factors, alcoholic chronic hepatic disease (Child-Pugh B) with thrombocytopenia, and severe calcified aortic stenosis was admitted for an elective transcatheter aortic valve implantation (TAVI). After the procedure, the patient was hypotensive and unresponsive to fluid challenge, and there was a significant difference in blood pressure between the two arms. The echocardiogram confirmed the normal position and function of the prosthetic valve but was suggestive of aortic dissection. A thoracic angiotomography was urgently done and revealed a flap of the intima layer of the aorta at the aortic arch level, with a false lumen beginning proximal to the left subclavian artery but without extension to the ascending or descending aorta. A multidisciplinary team opted for conservative management of the aortic dissection, with medical treatment only. The patient was discharged later and remains clinically stable at a one-year follow-up.

## Introduction

Surgical aortic valve replacement (SAVR) has long been considered the gold standard treatment for symptomatic severe aortic stenosis (AS) in younger adults. This procedure provides durable results and symptom relief; however, it carries inherent surgical risks, particularly in patients with significant comorbidities [1]. In recent years, transcatheter aortic valve implantation (TAVI) has emerged as an effective, minimally invasive alternative for patients deemed high-risk or unsuitable for surgery [2]. TAVI has demonstrated favorable outcomes in elderly populations and those with prohibitive surgical risk, offering reduced recovery times and fewer perioperative complications [3]. Despite these advantages, TAVI is not without risks, including vascular complications, paravalvular leaks, conduction disturbances, and, in rare cases, aortic dissection [4].

As the indications for TAVI expand to include younger and lower-risk populations, understanding and mitigating its potential complications become increasingly important. While life-threatening complications are relatively rare, their occurrence underscores the necessity of meticulous procedural planning, careful patient selection, and vigilant post-procedure monitoring [5]. Aortic dissection during TAVI is a rare but serious complication. Predisposing factors include anatomical issues such as severe aortic calcification, increased aortic root diameter, porcelain aorta, small aortic annulus, and extensive atheromatous plaques. Procedural factors include valve oversizing, improper prosthesis positioning, excessive balloon pre- or post-dilation, aggressive catheter manipulation, and improper guidewire handling. Patient-related factors include connective tissue disorders (e.g., Marfan or Ehlers-Danlos syndromes), severe hypertension, advanced age, and a history of aortic dissection. Careful pre-procedural imaging and selection of the appropriate prosthesis are crucial to minimizing this risk.

This study presents a challenging scenario in which a patient with significant comorbidities experienced a severe complication following TAVI and was managed conservatively, emphasizing the importance of individualized treatment strategies.

## Case presentation

A 53-year-old female with a medical history of multiple cardiovascular risk factors, including hypertension, obesity, dyslipidemia, and alcoholic chronic hepatic disease (Child-Pugh B) with severe thrombocytopenia (38,000/μL) and restrictive ventilation syndrome, presented with progressive heart failure symptoms (classified in New York Heart Association {NYHA} class II-III) over the course of a few months, including dyspnea on mild exertion, orthopnea, and fatigue. The ECG was normal; however, echocardiography revealed severe calcified aortic stenosis with preserved biventricular ejection fraction. Coronary angiography confirmed the absence of obstructive coronary artery disease. Due to the patient’s hepatic disease with thrombocytopenia, pulmonary disease, and associated surgical risks (Society of Thoracic Surgeons {STS} score 5.92%), she was deemed unsuitable for SAVR and TAVI was selected as the preferred treatment option.

The elective TAVI procedure was performed using a balloon-expandable aortic bioprosthesis valve (SAPIEN 3; Irvine, CA: Edwards Lifesciences) and was initially successful. However, the patient experienced complications right upon admission to the intensive care unit, minutes after the procedure. She became obnubilated, hypotensive, and unresponsive to fluid resuscitation, with a notable blood pressure differential between her arms (blood pressure in the right arm was 90/50 mmHg and in the left arm 65/30 mmHg). ECG findings revealed sinus bradycardia with de novo right bundle branch block and left anterior fascicular block. Urgent echocardiography confirmed the normal position and function of the prosthetic valve but raised concerns about aortic dissection, revealing a flap of the intima at the level of the aortic arch, with the false lumen having a maximum size of 9 mm in diastole (Video 1).

**Video 1:** Transthoracic echocardiogram (suprasternal view). Arrow pointing to intima flap of the aorta at aortic arch level, with the false lumen measuring a maximum of 9 mm in diastole.

Computed tomography angiography (CTA) confirmed the presence of an intimal flap consistent with dissection in the aortic arch (with a 9 mm false lumen originating proximally to the left subclavian artery but without extension to the ascending aorta, descending aorta, or the supra-aortic branches) (Video 2).

**Video 2:** Chest CT angiography. Arrow pointing to aortic dissection at aortic arch level. CTA confirmed the presence of an intimal flap consistent with dissection in the aortic arch (with a 9 mm false lumen originating proximally to the left subclavian artery but without extension to the ascending aorta, descending aorta or the supra-aortic branches). CTA: CT angiography

Given the high surgical risk of a non-A non-B aortic dissection, a multidisciplinary heart team decided on conservative management with medical treatment alone. During hospitalization, the patient developed a third-degree atrioventricular block, necessitating transvenous pacemaker implantation [6]. Follow-up echocardiography and CTA at 48 hours demonstrated no progression of the dissection. The patient progressed favorably with supportive medical treatment and was discharged nine days later, clinically well and asymptomatic. She remains clinically stable at a one-year follow-up and the dissection showed no signs of progression on the follow-up CT scan.

## Discussion

TAVI is increasingly being adopted as a viable alternative to SAVR for the treatment of severe symptomatic aortic stenosis, particularly in patients with significant comorbidities who present a high surgical risk [7]. In the present case, given the patient’s hepatic disease and high surgical risk, a non-surgical approach was chosen, emphasizing close hemodynamic monitoring and medical therapy. While TAVI generally results in favorable clinical outcomes, it is not devoid of complications. Vascular access complications, conduction abnormalities, and prosthetic valve dysfunction are commonly reported [8]. In rare instances, aortic dissection, as observed in our patient, can occur and presents a significant management challenge.

Type A aortic dissection, which involves the ascending aorta, is typically considered a surgical emergency due to the high risk of catastrophic outcomes, including aortic rupture and cardiac tamponade [9]. However, in patients with significant comorbidities or poor surgical candidacy, conservative management may be a reasonable alternative [10].

Aortic dissections limited to the aortic arch, or originating as retrograde dissections from the descending aorta that extend into the arch and stop before the ascending aorta, are termed as non-A non-B aortic dissection, as seen in the present case. Non-A and non-B dissection patients tend to be younger (median age 59 years) and have a lower mortality than acute type A aortic dissection. The 30-day mortality rate in patients treated medically is around 14%, compared to 4.4% in those successfully treated surgically. Conservative management leads to high mortality (malperfusion, aortic rupture). Thus, surgery or endovascular therapy is favored within 14 days of symptom onset. For complicated non-A and non-B aortic dissections with an arch tear, consider Frozen Elephant Trunk (FET) repair. However, if feasible, stent-graft implantation for primary tear coverage is an alternative [11]. Although this is the treatment recommended by the latest guidelines, a conservative approach was chosen for the patient in this case, as previously explained, which proved to be a prudent and successful decision.

The development of third-degree atrioventricular block requiring pacemaker implantation further highlights the procedural complexities of TAVI. Conduction disturbances are a recognized complication of TAVI, often attributed to the anatomical proximity of the conduction system to the aortic valve annulus [12]. This underscores the importance of thorough pre-procedural imaging and intra-procedural monitoring to anticipate and address such complications promptly.

## Conclusions

This case underscores the potential complications associated with TAVI, particularly in high-risk patients with significant comorbidities. Aortic dissection and conduction disturbances represent severe but manageable complications when identified and treated appropriately. The conservative approach adopted in this case highlights the importance of individualized patient management, balancing procedural risks with the patient’s overall condition and surgical suitability.

Long-term follow-up remains crucial to ensure the continued stability of such patients and to optimize outcomes. As TAVI continues to evolve and be utilized in broader patient populations, ongoing vigilance, patient selection refinement, and improved procedural techniques will be essential to minimize risks and enhance clinical success.
